# Dyslipidaemia and Intima-Media Thickness of Carotid Arteries in Thirty-Five HIV/AIDS Patients Receiving Highly Active Antiretroviral Therapy

**Published:** 2009-06

**Authors:** Xuexiang Zhang, Xueyan Jiang, Hongzhou Lu, Fang Shen, Jiangrong Wang

**Affiliations:** *Shanghai Public Health Clinical Center, Fudan University, Shanghai, China*

**Keywords:** highly active antiretroviral therapy, dyslipidaemia, intima-media thickness of carotid artery

## Abstract

**Objectives:**

Highly active antiretroviral therapy (HAART) has being impacted significantly therapies for natural human immunodeficiency virus (HIV) infection, which leads to a remarkable decrease in its morbidity and mortality but it is frequently associated with metabolic complications such as dyslipidaemia and cardiovascular complications. HIV reverse transcriptase (RT) inhibitors can be classified into nucleoside and non-nucleoside types. Mitochondrial dysfunction due to the depletion of mt-DNA is partly responsible for various nucleoside RT inhibitors-associated adverse effects including dyslipidaemia. Efavirenz (EFV) is metabolized primarily by cytochrome P450 2B6 (CYP2B6) and the metabolic effects of EFV have been described previously. All patients in this study received the same HAART treatment regime (Stavudine (d4T) + Lamivudine (3TC) + Efavirenz (EFV)). This study aims to assess incidences for dyslipidaemia and atherosclerosis.

**Methods:**

This retrospective study was conducted within outpatients of Shanghai Public Health Center. We selected thirty-five HIV-1 infected patients who receiving highly active antiretroviral therapy. Their mean CD4 cell count was 69.5 (±34.6) copies per micro liter before therapy. Fasting total cholesterol, triglyceride, high-density lipoprotein (HDL) cholesterol and low-density lipoprotein (LDL) cholesterol values were respectively compared among patients at present and before therapy. Then the data was statistically analyzed. Twenty-two patients had the intima-media thickness (IMT) of their carotid arteries measured by Philips 5000 Color-Doppler ultrasound tests. Results: After therapy, patients achieved significant changes in levels of triglycerides (1.44 ± 0.35mmol/L Vs. 2.07 ± 0.54mmol/L) (*P*<0.001), total cholesterol (4.96 ± 0.46 mmol/L Vs. 6.15 ± 0.83mmol/L) (*P*<0.001) and LDL cholesterol (2.29 ± 0.33 Vs. 3.11 ± 0.29 mmol/L) (*P*<0.001). In contrast, the level of HDL cholesterol did not significantly change (1.06 ± 0.01 mmol/L Vs. 1.04 ± 0.01 mmol/L) (*P*>0.5). The mean IMT of twenty-two patients was (0.86 ± 0.14) mm after HAART, which is higher than the norm age-matched value of (0.7 ± 0.2) mm (*P*<0.05).

**Results:**

After therapy, patients achieved significant changes in levels of triglycerides (1.44 ± 0.35mmol/L Vs. 2.07 ± 0.54mmol/L) (*P*<0.001), total cholesterol (4.96 ± 0.46 mmol/L Vs. 6.15 ± 0.83mmol/L) (*P*<0.001) and LDL cholesterol (2.29 ± 0.33 Vs. 3.11 ± 0.29 mmol/L) (*P*<0.001). In contrast, the level of HDL cholesterol did not significantly change (1.06 ± 0.01 mmol/L Vs. 1.04 ± 0.01 mmol/L) (*P*>0.5). The mean IMT of twenty-two patients was (0.86 ± 0.14) mm after HAART, which is higher than the norm age-matched value of (0.7 ± 0.2) mm (*P*<0.05).

**Conclusion:**

These data suggest that HAART is potentially dangerous for hyperlipidaemia and maybe an increase in atherosclerosis.

## INTRODUCTION

Acquired Immune Deficiency Syndrome (AIDS) has become one of the serious challenges in China. As of the end of September 2008, China has an accumulative report of more than 260 thousand cases for HIV-infection, of which 77 thousand cases are AIDS patients and death cases reach 34 thousand. In recent years, the opportunistic infections associated with AIDS have been reduced significantly by virtue of Highly Active Antiretroviral Therapy (HAART). However, dis-regulations on endocrine system and metabolism in patients receiving HAART have been reported frequently (1). HAART drugs such as protease inhibitors, nucleoside reverse transcriptase inhibitors (NRTIs) and their combined usage usually result in fat metabolism syndrome, which is one of relatively common long-term adverse effects with an average incidence rate of 50% (11%~83%) during treatments for AIDS patients. Hypertriglyceridemia and hypercholesterolaemia are associated with PI-based antiretroviral therapy (2). Additionally, dyslipidaemia is also reported relative to Stavudine and non-nucleoside reverse transcriptase inhibitors (NNRTIs) (3). The risk for cardiovascular complications can be predicted by intima-media thickness (IMT), which reveals the lipid accumulation in arteries and can be tested by Color-Doppler ultrasound (4). In order to explore HAART impact on blood lipid levels and cardiovascular complications, a retrospective study was conducted in our Center to assess dyslipidaemia and IMT in 35 patients who received HAART from Oct 2001 to Sept 2005.

## DATA AND METHODS

### Normal data

All 35 patients were confirmed in Shanghai Center for Disease Control and Prevention and initiated medications according to AIDS Diagnosis Standard issued by the American Centers for Disease Control and Prevention (US-CDC). They all received the same HAART regime (d4T+3TC+ EFV) in our Center as well as a long-term follow-up. The mean age was 38.5 (±15.3), mean length of therapy was 24.6 (±17.5) months from Sept 2001 to Sept 2005 and mean CD4 cell count was 69.5 (±34.6) copies per micro liter before therapy.

### Methods for blood lipid level and IMT test

Fasting blood lipids of every patient pre-therapy and post-therapy were tested: TC by the CHOD-PAP method and TG by the GPO-PAP method; HDL and LDL were tested by deposition. The coefficients of variation for cholesterol and triglyceride values are within 3% and 5%, respectively. Of all patients 22 received Philips 5000 Color-Doppler ultrasound voluntarily in the last assessment in the study.

### Standards for diagnosis

Chinese standard for hyperlipidaemia diagnosis was issued in 1997 (5). Total TC level less than 5.20 mmol/L (200 mg/dL) is defined as normal; and that greater than 6.2 mmol/L (240 mg/dL) is diagnosed as hypercholesterolemia. TG level less than 1.70 mmol/L (150 mg/dL) is defined as normal; and that greater than 2.3 mmol/L (200 mg/dL) is diagnosed as hypertriglyceridemia. HDL-C level greater than 1.0 mmol/L (40 mg/dL) is defined as normal.

### Statistical analysis

Data were assessed by mean ± standard deviation and analyzed with SPSS10.0 statistical analysis software. It will be was considered significant for *P*<0.05. Additionally, single-factor analysis was applied to the blood lipids analysis before and after antiretroviral therapy and t-test was used to compare the mean figures.

## RESULTS AND CONCLUSIONS

### Blood lipid changes (Table 1)

- Triglyceride: There was significant difference between triglyceride levels before and after HAART (*P*<0.001). The mean triglyceride level of 35 patients before therapy (d4T + 3TC + EFV) was 1.44 ± 0.35 mmol/L, which was less than the normal level according to diagnosis standard; but it rose to 2.07 ± 0.54 mmol/L exceeding the normal level at the end of this study.
- Total cholesterol: There was also significant change between cholesterol levels before and after HAART (*P*<0.001). The mean level was 4.96 ± 0.46 mmol/L before therapy, which increased to 6.15 ± 0.83 mmol/L during therapy.
- HDL: HDL cholesterol did not significantly change (*P*>0.5), being 1.06 ± 0.01 mmol/L before therapy and 1.04 ± 0.01mmol/L after.
- LDL: LDL cholesterol changed significantly (*P*<0.001) from 2.29 ± 0.33 mmol/L to 3.11 ± 0.29 mmol/L.

**Table 1:** Changes of mean blood lipids before and after HAART

	Pre-therapy	Post-therapy	*P*

TG (mmol/L)	1.44 ± 0.35	2.07 ± 0.54	P<0.001
TC (mmol/L)	4.96 ± 0.46	6.15 ± 0.83	P<0.001
HDL (mmol/L)	1.06 ± 0.01	1.04 ± 0.01+	P>0.05
LDL (mmol/L)	2.29 ± 0.33	3.11 ± 0.29	P<0.001

### Intima-media thickness of carotid artery

22 patients received IMT test by ultrasound, and the mean value was (0.86 ± 0.14) mm, which was thicker than the normal value in age-matched controls (0.7 ± 0.2) mm (*P*<0.05) (2, 6).

## DISCUSSIONS

It is known that HAART can reduce the complications of AIDS and decrease the morbidity and mortality dramatically. However, it is also associated with therapy-related dyslipidaemia and lipodystrophy. It was found in this study that patients receiving HAART therapy of d4T, 3TC & EFV are potentially dangerous for dyslipidaemia. HIV RT inhibitors can be classified into nucleoside and non-nucleoside RT inhibitors. Nucleoside RT inhibitors (NRTI, such as d4T) are converted to active triphosphate analogues and incorporated into DNA in RT-catalyzed reactions. They act as chain terminators for blocking DNA synthesis since they lack the 3′-OH group required for the phosphor-diester bond formation. Mitochondrial dysfunction due to the depletion of mt-DNA is partly responsible for various NRTI-associated adverse effects including myopathy, cardiomyopathy, peripheral neuropathy, pancreatitis, hepatic steatosis, lipodystrophy and lactic acidosis in severe cases (12). It has been shown in recent researches that patients with d4T are prone to hypercholesterolemia and hypertriglyceridemia. Efavirenz (EFV) is metabolized primarily by cytochrome P450 2B6 (CYP2B6) and high plasma concentrations of this drug are associated with a G-->T polymorphism at position 516 (516G-->T) of CYP2B6 and frequent central nervous system (CNS)-related side effects. The metabolic effects of EFV have been described previously and some studies have shown that EFV might be associated with dyslipidaemia (14). The significance of the dyslipidaemia observed with these and other antiretroviral agents remains unknown. Some studies have found no association between HAART and an increased cardiovascular risk, and others have documented an increase in cardiovascular disease associated clinical events (13). At present it seems more studies report an increase in cardiovascular disease than the opposite, so these changes induced by HAART should probably be taken seriously. Clinicians should monitor for and treat hyperlipidemia in their patients to lesson these possible HAART associated complications. The tremendous decrease in death rate associated with the use of HAART in HIV disease surely outweighs concerns about possible small increases in cardiovascular disease, which can already be found commonly in general population. However, this risk seems real and should be monitored for and treated to reduce any impact it may have.

Detection of increased IMT of carotid arteries by ultrasound can predict atherosclerotic coronary artery disease (7, 8). It is reported that injury is more likely to happen in target organs when the IMT is greater than 0.9 mm (9). The mean IMT in this study was greater than the norm value (2), which suggested that some of our patients are at risk of cardiovascular complications at that time (9-11). Some patients in our study have in fact had clinically significant atherosclerotic coronary artery disease. However, not all patients in the study were tested for IMT and pre-therapy data was absent. The contribution of HIV disease itself to possible increasing on atherosclerosis is difficult to be separated from that of HAART. More and prospective studies and those carrying out in other countries would be more useful.

## References

[1] Das S (2005). HIV and increased risk of cardiovascular diseases. Sex Health.

[2] Zareba KM, Miller TL, Lipshultz SE (2005). Cardiovascular disease and toxicities related to HIV infection and its therapies. Expert Opin. Drug Saf.

[3] Young J, Weber R, Rickenbach M, Furrer H (2005). Lipid profiles for antiretroviral-naive patients starting PI- and NNRTI-based therapy in the Swiss HIV cohort study. Antivir Ther.

[4] Umeh OC, Currier JS (2005). Lipids. Metabolic syndrome, and risk factors for future cardiovascular disease among HIV-infected patients. Curr. HIV/AIDS Rep.

[5] Oh J, Hegele RA (2007). HIV-associated dyslipidaemia: pathogenesis and treatment. Lancet Infect. Dis.

[6] Spieker LE, Karadag B, Binggeli C (2005). Rapid progression of atherosclerotic coronary artery disease in patients with human immunodeficiency virus infection. Heart Vessels.

[7] Fultz SL, Goulet JL, Weissman S (2005). Differences between infectious diseases-certified physicians and general medicine-certified physicians in the level of comfort with providing primary care to patients. Clin. Infect. Dis.

[8] Jones R, Sawleshwarkar S, Michailidis C (2005). Impact of antiretroviral choice on hypercholesterolaemia events: the role of the nucleoside reverse transcriptase inhibitor backbone. HIV Med.

[9] Calza L, Colangeli V, Manfredi R (2005). Rosuvastatin for the treatment of hyperlipidaemia in HIV-infected patients receiving protease inhibitors: a pilot study. AIDS.

[10] Calza L, Manfredi R, Colangeli V (2005). Substitution of nevirapine or efavirenz for protease inhibitor versus lipid-lowering therapy for the management of dyslipidaemia. AIDS.

[11] Sekhar RV, Jahoor F, Pownall HJ (2005). Severely dysregulated disposal of postprandial triacylglycerols exacerbates hypertriacylglycerolemia in HIV lipodystrophy syndrome. Am. J. Clin. Nutr.

[12] Akihiko Saitoh, Richard H. Haas, Robert K, and Naviaux (2008). Impact of Nucleoside Reverse Transcriptase Inhibitors on Mitochondrial DNA and RNA in Human Skeletal Muscle Cells. Antimicrob Agents Chemother.

[13] Kotler DP (2008). HIV and antiretroviral therapy lipid abnomilities and associated caidiovascular risk in HIV-infected patients. J. Acquir. Immune. Defic. Syndr.

[14] Bain AM, White EA, Rutherford WS, Rahman AP (2008). A multi-modal, evidence-based approach to achieve lipid targets in the treatment of antiretroviral-associated dyslipidemia: case report and review of the literature. Pharmacotherapy.

